# Elderly nasopharyngeal carcinoma patients (aged ≥70 years): Survival and treatment strategies

**DOI:** 10.1002/cam4.6562

**Published:** 2023-09-19

**Authors:** Gang Yang, Jingjing Huang, Ji Sun, Li Wang

**Affiliations:** ^1^ Department of Radiotherapy, Eye Ear Nose and Throat Hospital, Shanghai Medical College Fudan University Shanghai PR China; ^2^ ENT institute and Department of Otorhinolaryngology, Eye & ENT Hospital Fudan University Shanghai PR China; ^3^ Department of Pathology, Eye & ENT Hospital Fudan University Shanghai PR China

**Keywords:** chemotherapy, elderly, IMRT, nasopharyngeal carcinoma, survival outcomes

## Abstract

**Background:**

With the coming of the aging society, the incidence of elderly nasopharyngeal carcinoma (NPC) has been increasing which may result in considerable disease burden; however, the optimal treatment strategy for elderly patients is still debatable.

**Methods and Results:**

Clinical data on 294 elderly NPC patients aged ≥70 treated between 2009 and 2019 was analyzed. Kaplan–Meier method was used to estimate overall survival (OS) and cancer‐specific survival (CSS) rates. With a median follow‐up of 53.25 months, the 5‐year estimated OS and CSS for the entire group were 59.5% and 69.8%, respectively. 146 patients died within the follow‐up period, of which recurrence + metastasis (48%) and internal medical disease unrelated to NPC (32%) are the primary causes of death. On univariable analysis, (IMRT vs. 3D‐CRT) (*p* = 0.001; *p* = 0.000), T stage (*p* = 0.001; *p* = 0.000), N stage (*p* = 0.013; *p* = 0.000) and clinical stage (*p* = 0.000; *p* = 0.000) were associated with OS and CSS; Charlson Comorbidity Index (CCI) (*p* = 0.016) was associated with OS. The addition of chemotherapy (CT) correlated with better CSS (*p* = 0.039), but did not improve OS (*p* = 0.056) for stage III–IV subgroup. On multivariate analysis, advanced clinical stage independently predicted poorer OS (*p* = 0.002) and CSS (*p* = 0.000). In addition, the application of IMRT was an independent protective factor on both OS (*p* = 0.028) and CSS (*p* = 0.030).

**Conclusion:**

IMRT is a reasonable treatment strategy to improve survival for elderly NPC patients aged over 70 years; consideration of adding chemotherapy for elderly population should be weighed carefully.

## 1 INTRODUCTION

Nasopharyngeal carcinoma (NPC) is an epithelial carcinoma and endemic mainly in southern China and Southeast Asia, with a single peak at approximately ages 45–59 years and beginning to decline at ages over 60 years.^1^, ^2^ It is highly sensitive to ionizing radiation and a radiation dose of 66–70 Gy is required.^3^ Currently, radiotherapy (RT) with or without chemotherapy (CT) has been the mainstay treatment modality for newly diagnosed NPC. Furthermore, with combined use of magnetic resonance imaging (MRI) staging, intensity‐modulated radiation therapy (IMRT), and concurrent chemoradiotherapy, the survival outcomes of NPC has substantially improved over the past two decades; moreover, the 5‐year overall survival (OS) rate has already reportedly been over 85% in locoregionally advanced NPC patients aged 18–59 years.^4^, ^5^ However, the treatment guidelines of NPC are often based on clinical studies that, generally, exclude patients who are ≥70 years of age due to high comorbidity burden and suboptimal organ function.^6^, ^7^ Thus, whether the conclusions from these studies could apply to elderly individuals aged over 70 years remains controversial.

With the coming of the aging society, the incidence of elderly NPC has be increasing which may result in considerable disease burden; about 10%–15% cases have their diagnosis at age ≥ 70.^8^ Elderly NPC patients are often accompanied with multiple co‐morbid conditions and more likely to suffer severe chemoradiotherapy‐related toxicity in comparison to younger individuals, so the treatment strategy may be more complex and difficult for the elderly. Several retrospective studies showed that elderly NPC patients aged ≥70 years had significantly poor survival outcomes, with the 5‐year OS rate range from 43.9% to 61.8%.^9^, ^10^, ^11^ At present, no standard of treatment in the elderly NPC patients was recommended for the insufficient evidence. We performed this study to investigate the optimal strategy and survival outcome in the elderly NPC patients aged ≥70 years.

## 2 METHODS

### 2.1 Patients

We retrospectively enrolled 294 patients aged ≥70 years with NPC who underwent RT at our hospital between January 2009 and October 2019. All the patients were confirmed by pathology. The demographics, disease characteristics, and follow‐up of the patients were obtained from the medical records. This study was compliant with ethical standards and was approved by the institutional ethics; all the patients provided written informed consent, allowed treatment data for research purposes and statistical analysis. For this study, all the cases were restaged with the 8th edition AJCC classification system. Pretreatment comorbidities data were extracted from the patients' medical chart, and the Charlson Comorbidity Index (CCI) score scoring system is shown in Table S1.

### 2.2 Treatment

All patients completed RT with or without CT. The total irradiated dose for primary nasopharyngeal tumor was 66–75.4 Gy; The RT techniques of three‐dimensional conformal RT (3D‐CRT) (102 patients) or IMRT (192 patients) was conducted for all patients once a day for five times a week.

For CT, the addition of induction, concurrent or adjuvant CT was based on physicians’ decision. 1–6 cycles of platinum‐based CT was prescribed for 175 patients. The patients were divided into RT‐alone group and radiotherapy + chemotherapy (RT + CT) group according to CT or not. The CT regimens used include gemcitabine (1 g/m^2^ d1,8) + cisplatin (20–25 mg/m^2^ d1‐3) or docetaxel (60–70 mg/m^2^ d1) + cisplatin (20–25 mg/m^2^ d1‐3) + 5‐fluorouracil (500–700 mg/m^2^ d1‐5) or cisplatin (20–25 mg/m^2^ d1‐3) + 5‐fluorouracil (500–700 mg/m^2^ d1‐5) repeated every 3 weeks.

### 2.3 Follow‐up

Each patient was followed up with a complete physical examination, blood test, and imaging studies (e.g., nasopharynx MRI with contrast, chest CT, abdominal ultrasound scan, and bone scanning in necessity) every 3 months for the first 2 years, every 6 months for the next 3 years, and yearly thereafter. The primary objective for analysis were 5‐year OS and cancer‐specific survival (CSS). Survival outcomes were calculated from the date of starting RT.

### 2.4 Statistical analysis

Treatment outcomes were analyzed by univariate and multivariate survival analyses for the patients (*n* = 294) using Log‐rank and Cox regression testing in SPSS statistical software version 26.0® (IBM). OS and CSS rate were calculated using the Kaplan–Meier method. Two‐tailed *p* < 0.05 were considered statistically significant.

## 3 RESULTS

### 3.1 Baseline characteristics

Patients' characteristics are displayed in Table 1. The median age of patients was 73 years (range, 70–88 years), and 69.7% (205/294) of the cases were men; 16.0% (47/294) of the patients had family history of cancer and 3.1% (9/294) had family history of NPC. The comorbidity level was sorted according to the CCI. 88.8% (261/294) of the cases were sorted ≤2. In total, there were 55.1% (162/294) patients with stage III–IV disease; T category distribution was: T1–2 (45.9%, 135/294) and T3–4 (54.1%, 159/294); N category distribution was: N0–1 (56.5%, 166/294) and N2–3 (43.5%, 128/294); six patients (2.0%) had distant metastasis. The prescribed dose to the primary nasopharyngeal tumor was >68 Gy (Median: 70.0 Gy, range from 68.8Gy to 75.4Gy) for 177(60.2%) patients and ≤ 68Gy (Median: 67.5 Gy, range from 66 to 68 Gy) for 117 (39.8%) patients, respectively. 175 (59.5%) patients were given platinum‐based CT.

**TABLE 1:** Patients' clinical characteristics.

Characteristic	*n* = 294 (%)
Age, years	
Median (range), years	73 (70–88)
<75(%)	182 (61.9)
≥75(%)	112 (38.1)
Gender (%)	
Male	205 (69.7)
Female	89 (30.3)
Tumor family history (%)	
No	247 (84.0)
Yes	47 (16.0)
NPC family history(%)	
No	285 (96.9)
Yes	9 (3.1)
CCI (%)	
≤2	261 (88.8)
>2	33 (11.2)
T stage (%)	
T1–2	134 (45.6)
T3–4	160 (54.4)
N stage (%)	
N0–1	166 (56.5)
N2–3	128 (43.5)
M stage (%)	
M1	6 (2.0)
M0	288 (98.0)
Clinical stage (%)	
I–II	132 (44.9)
III–IV	162 (55.1)
RT dose (%)	
66–68 Gy	117 (39.8)
>68 Gy	177 (60.2)
RT technique (%)	
3D conformal RT	102 (34.7)
IMRT	192 (65.3)
Chemotherapy (%)	
Yes	175 (59.5)
No	119 (40.5)

### 3.2 Survival and death analysis

**FIGURE 1:**
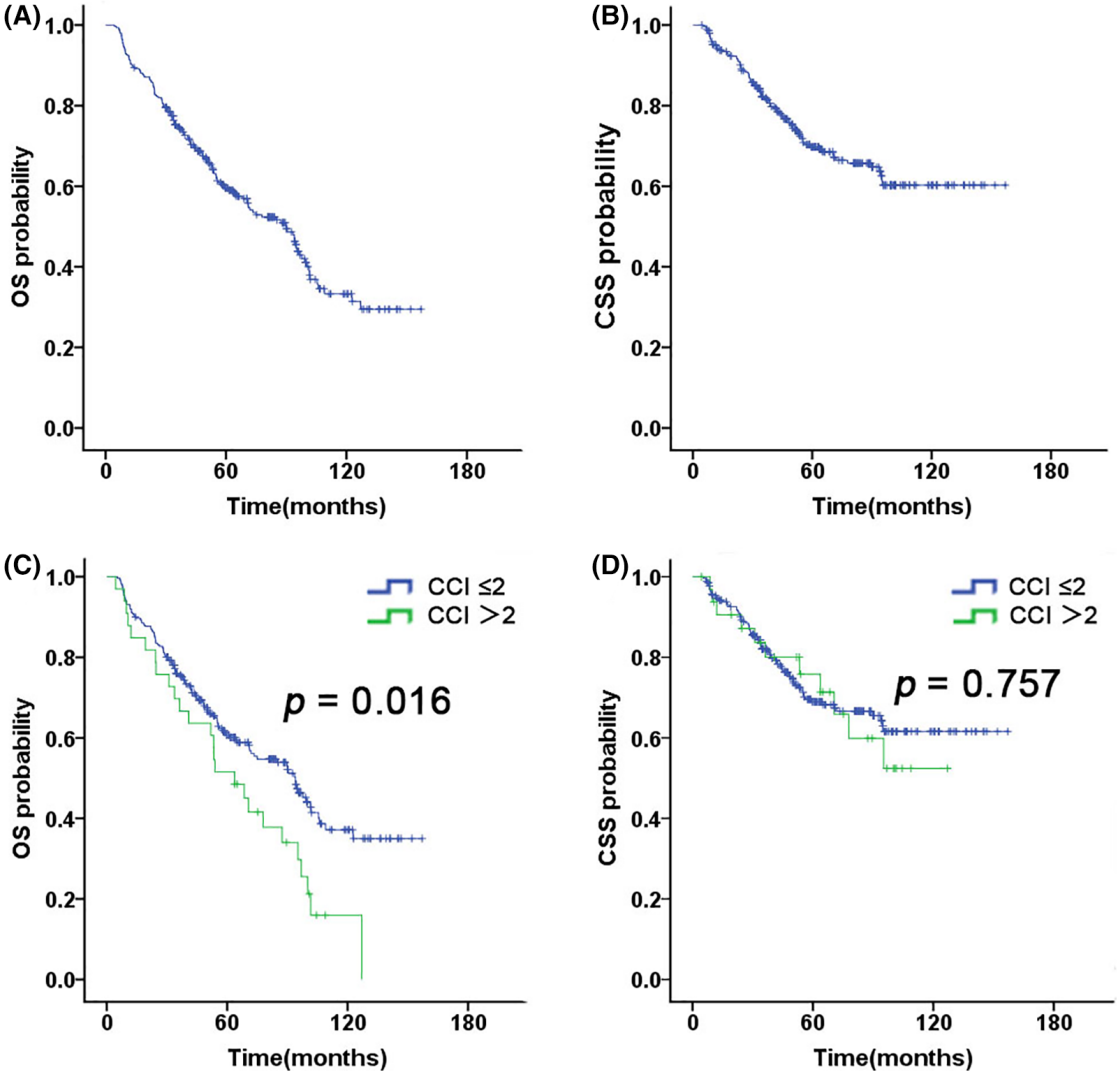
Kaplan–Meier estimates of (A) overall survival (OS), (B) cancer‐specific survival (CSS) for elderly NPC patients aged ≥ 70; Kaplan–Meier estimates of (C) OS and (D) CSS on NPC patients stratified by CCI.

**FIGURE 2:**
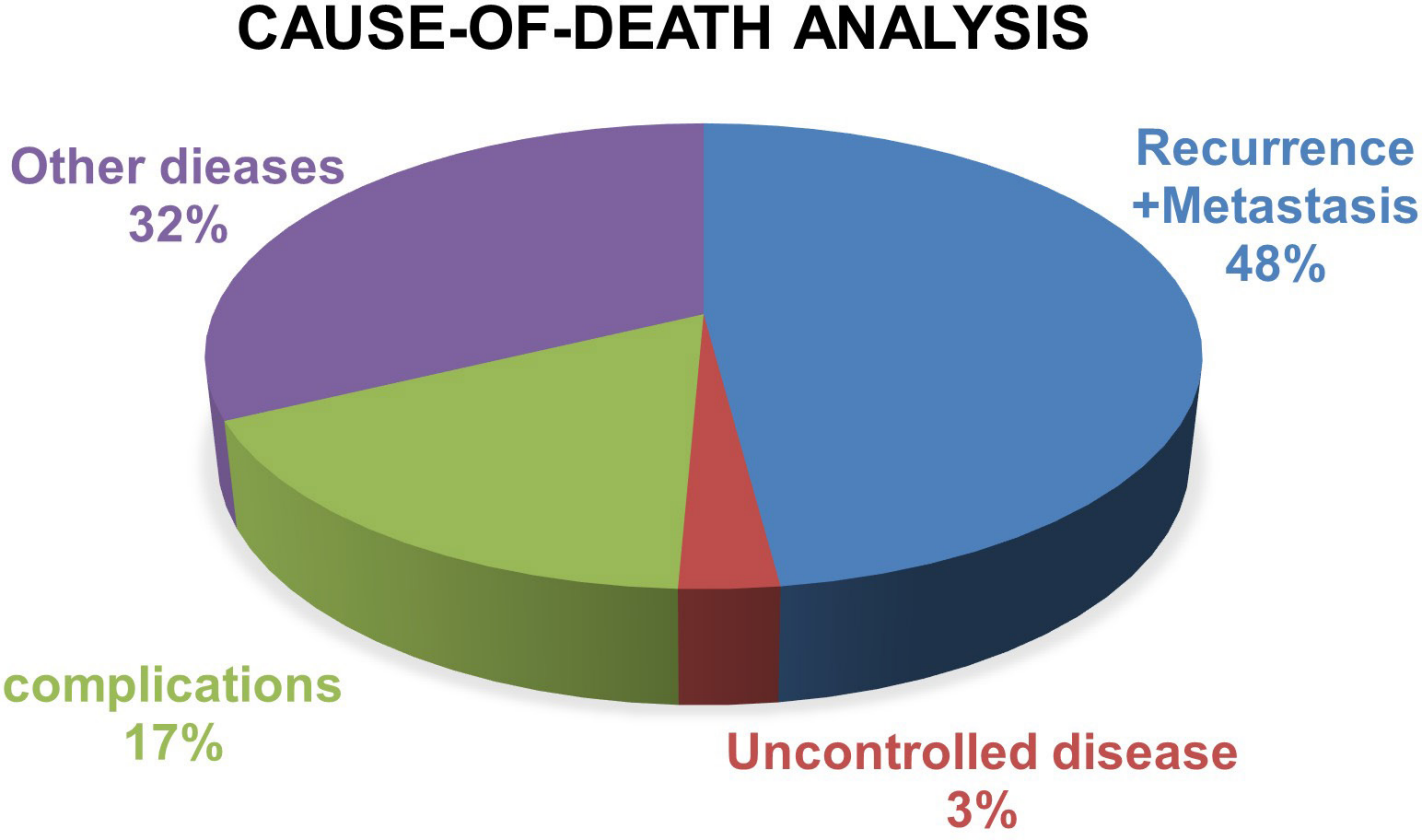
Cause of death analysis.

In this study, 148 patients were alive. The median follow‐up duration was 53.25 months (range, 4.5–157.1 months) for the entire cohort and 65.55 months (range, 14.2–157.1 months) among surviving cases, respectively. Besides, the 5‐year OS and CSS rates were 59.5% and 69.8%, respectively (Figure 1A,B). Of the 146 patients who died, 70 patients (48%) died of recurrence or metastasis, 47 (32%) died of internal medical disease unrelated to the chemoradiotherapy, 25 (17%) died of toxicities related to the chemoradiotherapy, and 4 patients (3%) died of uncontrolled tumor (Figure 2).

### 3.3 Prognostic factors

**FIGURE 3:**
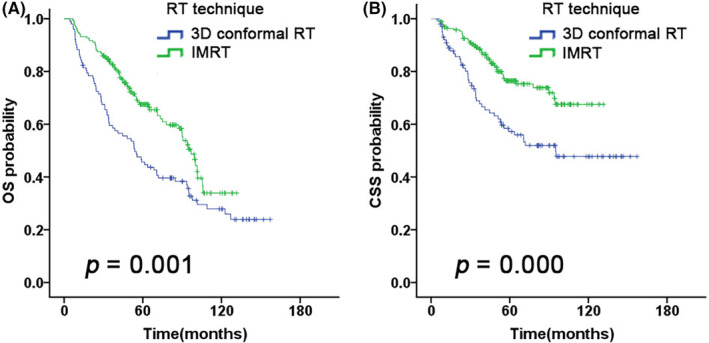
Kaplan–Meier estimates of (A) OS and (B) CSS on NPC patients stratified by RT technique.

**FIGURE 4:**
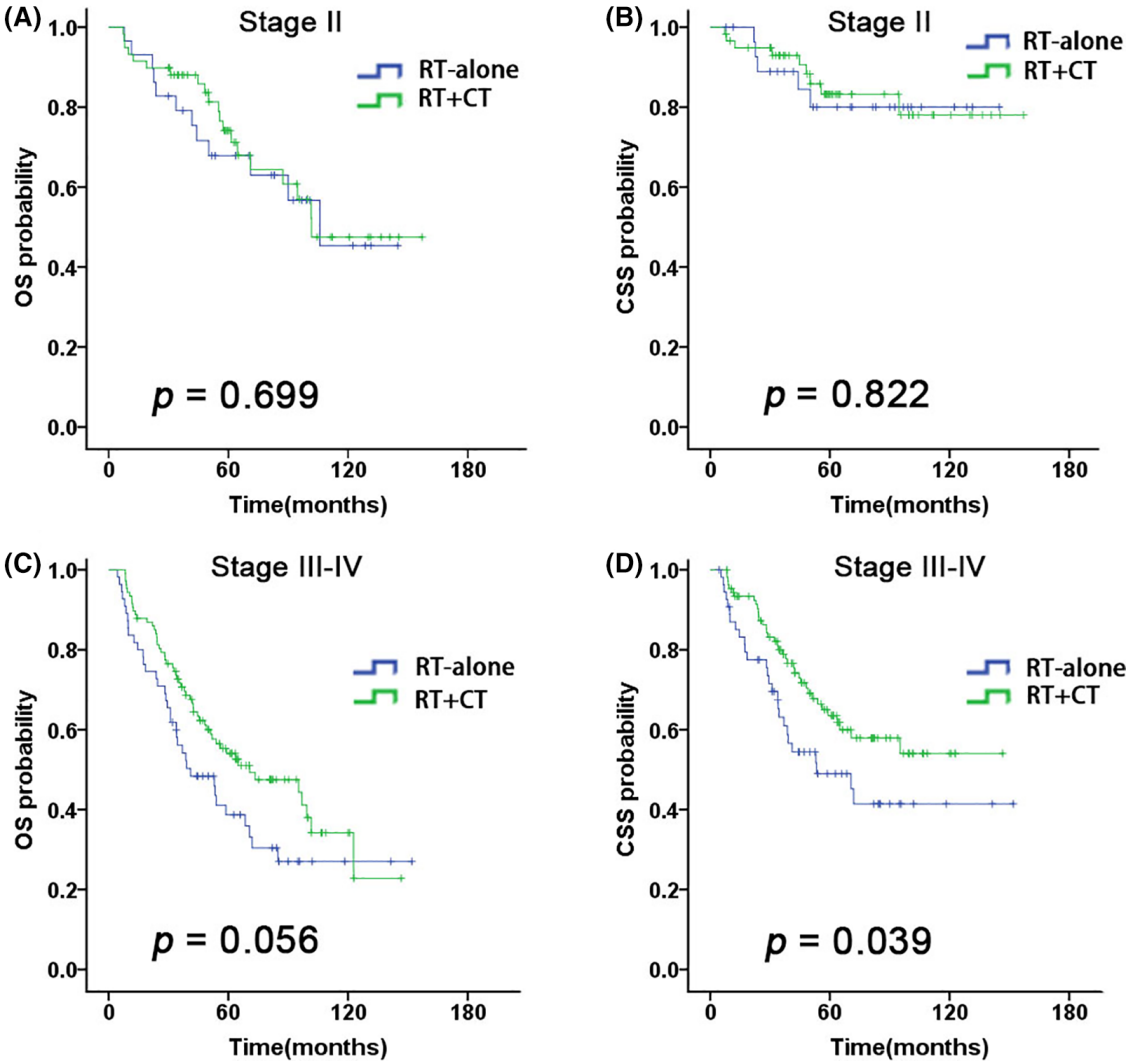
Kaplan–Meier analysis of (A) OS and (B) CSS is stratified by chemotherapy (CT) in stage II patients; Kaplan–Meier analysis of (C) OS and (D) CSS is stratified by chemotherapy (CT) in stage III–IV patients. RT‐alone: Radiotherapy alone; RT + CT: Radiotherapy + Chemotherapy.

We evaluated several potential prognostic factors including CCI, clinical stage, T and N stage, RT technique, and CT. On univariable analysis, CCI score (≤ 2 vs. > 2, *p* = 0.016) was significantly associated with OS (Figure 1C); RT technique (IMRT vs. 3D‐CRT, *p* = 0.001, *p* = 0.000), T stage (T1–2 vs. T3–4, *p* = 0.001, *p* = 0.000), N stage (N0–1 vs. N2–3, *p* = 0.013, *p* = 0.000) and clinical stage (I–II vs. III–IV, *p* = 0.000, *p* = 0.000) were significantly associated with OS and CSS (Figure 3, Figure S1). Besides, in the group of early‐stage (II) disease, CT did not show a survival advantage (RT + CT vs. RT‐alone, OS: *p* = 0.699; CSS: *p* = 0.834) (Figure 4A,B); in the group of advanced‐stage (III–IV) disease, CT was associated with superior 5‐year CSS rate (63.5% vs. 49.0%, *p* = 0.039), but did not improve the 5‐year OS rate (54.0% vs. 38.7%, *p* = 0.056) (Figure 4C,D).

Table 2 summarized the results of multivariate analyses on survivals. Advanced clinical stage (III–IV vs. I–II) was independently associated with poorer OS (HR, 1.759; 95% CI 1.233–2.509, *p* = 0.002) and CSS (HR, 2.705; 95% CI 1.646–4.444, *p* = 0.000). Besides, IMRT irradiation was a significant favorable prognostic factor for OS (HR, 0.676; 95% CI 0.477–0.958, *p* = 0.028) and CSS (HR, 0.556; 95% CI 0.354–0.874, *p* = 0.011).

**TABLE 2:** Multivariate analyses of factors in relation to OS and CSS using the cox proportional hazards model (*n* = 294).

Variables	OS			CSS		
	HR	95% CI	*p*	HR	95% CI	*p*
Age (≥75 vs. < 75)	0.996	0.711–1.394	0.981	1.078	0.700–1.660	0.734
Gender (Female vs. Male)	1.185	0.833–1.684	0.345	1.485	0.956–2.307	0.078
CCI(≤2 vs. >2)	1.521	0.991–2.336	0.055	0.930	0.491–1.761	0.824
Clinical stage (III‐IV vs. I–II)	1.759	1.233–2.509	**0.002**	2.705	1.646–4.444	**0.000**
RT technique (IMRT vs. 3D‐CRT)	0.676	0.477–0.958	**0.028**	0.556	0.354–0.874	**0.011**
CT (Yes vs. No)	0.865	0.611–1.225	0.413	0.886	0.563–1.394	0.601

*Note:* The bold values showed the clinical stage and radiotherapy technique are the significant prognostic factors for OS and CSS.

Abbreviations: CT: chemotherapy; CSS: cancer‐specific survival; HR: hazard ratio; OS: overall survival; RT: radiotherapy.

## 4 DISCUSSION

The individualized treatment for NPC patients over 70 years old is a neglected issue in clinical practice, as they are often excluded from most clinical trials that have tested therapeutic agents. However, with an aging population and an increasing number of elderly patients have been diagnosed with NPC (Figure S2), management of this group of patients, including their therapeutic efficacy and quality of life (QOL), will be increasingly important. In general, elderly NPC patients aged ≥70 have poor survival rates compared to the younger counterparts because of delayed diagnosis, advanced age, multiple comorbidities (such as cardiac disease), frailty, or poor performance status.^12^ While survival outcomes of NPC have significantly improved over the past decade, the treatment of the elderly population has always been a difficulty. So far, the optimal treatment strategy for elderly patients is debatable. To our knowledge, this retrospective study is the largest single centre dataset to explore the optimal treatment strategy and survival outcomes of elderly NPC patients aged over 70 years.

In accordance with the previous papers (5‐year OS, 43.9%–61.8%),^9^, ^10^, ^11^ our results showed acceptable survival outcome (5‐year OS, 59.5%; 5‐year CSS, 69.8%) in patients aged ≥ 70 years. The CCI has been reported to be a useful tool for predicting mortality in patients with cancers.^13^, ^14^, ^15^ In our data, CCI was significantly associated with OS (*p* = 0.016), but not with CSS (*p* = 0.757) in the univariate. Therefore, CCI is a useful tool to predict the treatment outcome of elderly patients which is of paramount importance for the management of elderly population aged ≥ 70 years since they are characterized by a higher burden of comorbidities compare to younger counterparts.

Cause‐of‐death analysis is particularly valuable for the treatment strategies studies of the elderly NPC population. In our analysis, totally 294 elderly patients with NPC, including 146 who died (49.7%, 146/294), were assessed. Nearly half of patients (48%, 70/146) died from recurrence and metastasis, whereas 32% (47/146) died of other diseases; and the most common noncancer cause of death were cardio‐cerebro‐vascular diseases and pneumonia. These findings could help guide physicians making clinical decision and taking care of elderly NPC patients.

RT is the standard treatment for newly diagnosed NPC. As a relatively new RT technique, IMRT significantly improves survival outcomes and reduces RT‐related adverse events in the past two decades.^16^, ^17^ In Cao et al.'s^18^ study, the application of IMRT resulted in a survival benefit for patients aged 65–79 years (5‐year CSS 69.4%). In our study, IMRT was associated with a better 5‐year OS (67.5% vs 44.6%, *p* = 0.001) and CSS (76.4% vs. 57.2%, *p* = 0.000) than was 3D‐CRT in elderly NPC patients aged ≥ 70 (Figure 3), which is consistent with other reports; furthermore, multivariate analyses showed a significant benefit in OS (*p* = 0.028) and CSS (*p* = 0.011) after IMRT (Table 2) that indicated the results of treating elderly patients aged ≥ 70 with NPC by IMRT were excellent.

Radiation doses are associated with efficacy and toxicity; radiation dosages of around 70 Gy to the primary tumor is considered the standard treatment in adult NPCs.^19^ However, high dose radiation is closely associated with severe acute and long‐term toxicities, especially for the elderly, which compromises their QOL. The study by Sze et al.^10^ indicated that patients aged ≥ 70 had poor tolerance to high‐dose RT with higher rates of RT‐related toxicities compared with the younger counterparts. De‐escalation of radiation dose has become an important research domain in improving QOL for cancer patients. A retrospective study by Wang et al.^20^ suggested that a moderately reduced dose (53‐67Gy) delivered with IMRT resulted in comparable outcomes to standard dose (70Gy) for T1–3 NPC; Xue et al.^21^ demonstrated that dose de‐escalated (reducing from 70.4 to 66 Gy) IMRT was related to improved 5‐year progression‐free survival and less toxicities for induction chemotherapy‐sensitive T3–4 NPC patients. At present, limited studies have been reported on dose de‐escalated in elderly NPC patients. There is no consensus on whether a moderately reduced dose would compromise long‐term survival compared with the standard dose (70Gy) in patients aged ≥ 70 years which warrants further prospective investigation.

Another concern is whether CT could further improve the clinical outcomes of the elderly population age ≥ 70. Conflicting results were reported in the literature. Lyu et al.^11^ showed that no remarkable differences in survival outcomes were found between the RT‐alone and the RT + CT group in the elderly patients age ≥ 70; Sommat et al.^22^ also found that the addition of CT did not improve the survival outcomes in older patients with advanced NPC (age ≥ 65). However, the study by Lu et al.^23^ showed that the addition of CT provided longer OS compared to RT alone for elderly patients aged over 65 years. In the current study, we comparatively analysis the prognoses of stage II–IV population receiving RT alone or together with CT subgroup analysis indicated that the addition of CT did not appear to provide a survival advantage in patients with stage II; for stage III–IV patients, CT could significantly improve the CSS (*p* = 0.039), but showed no benefit on OS (*p* = 0.056) (Figure 4). The reason for this finding may be that the advanced patients were not further stratified by CCI, organ function, performance status and EBVDNA. As we all know, the addition of CT was related to increased acute and late toxicity. Therefore, the CT regimens and dosages in elderly people with advanced NPC should be weighed carefully. And, tailored and less aggressive CT regimens for elderly population (age ≥ 70) should be considered.

This study has several limitations. First, it is limited by its retrospective nature and potential for selection bias. Second, chemoradiotherapy‐related toxicities were not evaluated in our study due to a lack of detail on chemoradiotherapy‐related side effects. Therefore, further prospective multicenter clinical trials are warranted.

## AUTHOR CONTRIBUTIONS

**Gang Yang:** Conceptualization (equal); data curation (equal); methodology (equal); writing – original draft (equal). **Jing‐Jing Huang:** Methodology (equal); supervision (equal); writing – review and editing (equal). **Ji Sun:** Conceptualization (equal); data curation (equal); writing – review and editing (equal). **Li Wang:** Conceptualization (equal); investigation (equal); supervision (equal); validation (equal); writing – original draft (equal); writing – review and editing (equal).

## CONFLICT OF INTEREST STATEMENT

None.

## ETHICS STATEMENT

This study was compliant with ethical standards and was approved by the institutional ethics.

## PATIENT CONSENT STATEMENT

All the patients provided written informed consent, allowed treatment data for research purposes and statistical analysis.

## Supporting information


Figure S1.

Figure S2.

Table S1.

## Data Availability

Data available on request.
