# Supplementary figures and images for: Elderly nasopharyngeal carcinoma patients (aged ≥70 years): Survival and treatment strategies
Source: Cancer Med. 2023 Sep 19;12(19):19523–9. doi: 10.1002/cam4.6562 (PMC10587980; doi:10.1002/cam4.6562)

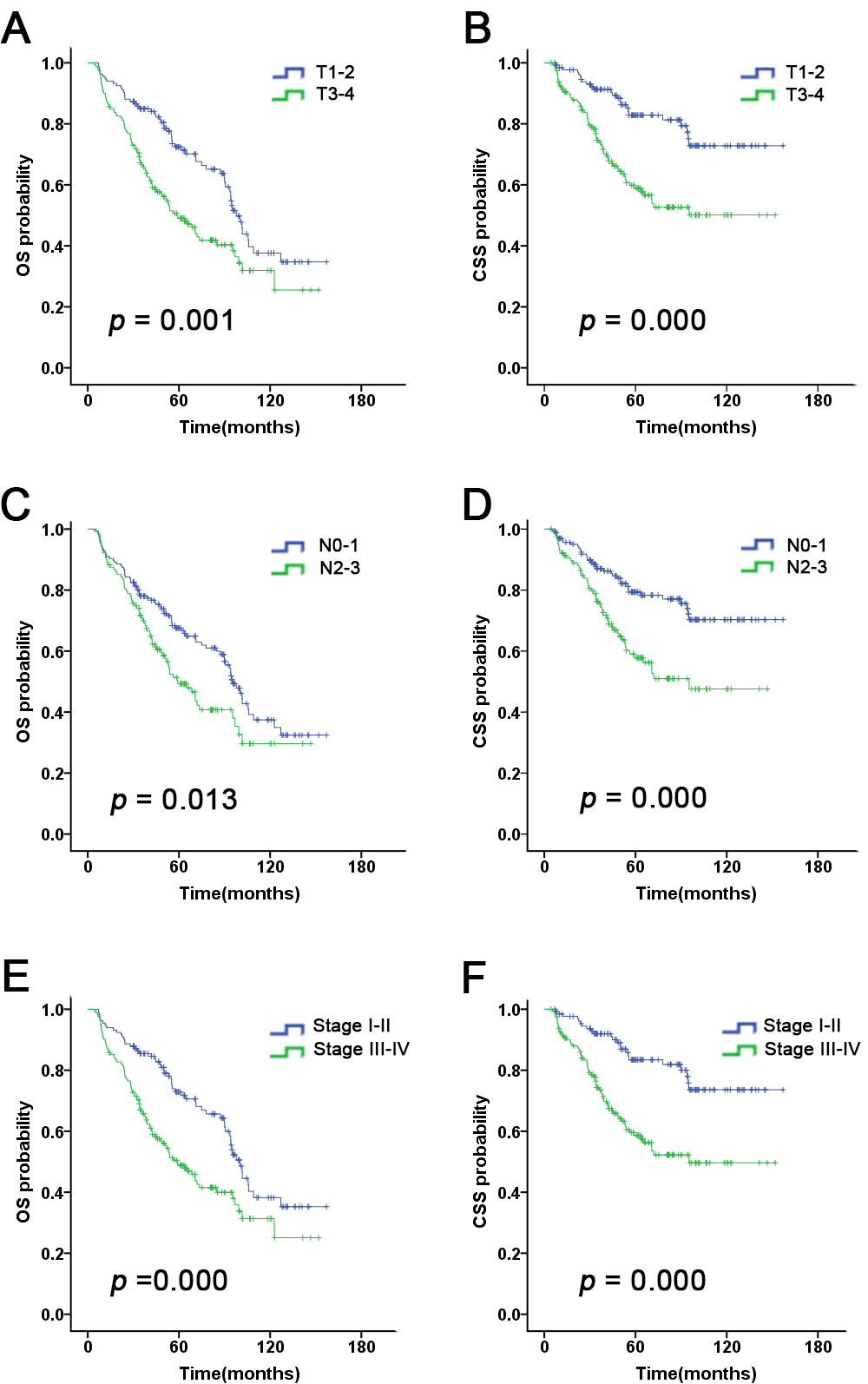

Supplement: Supplementary file 1 — Figure S1. [file CAM4-12-19523-s003.jpg]

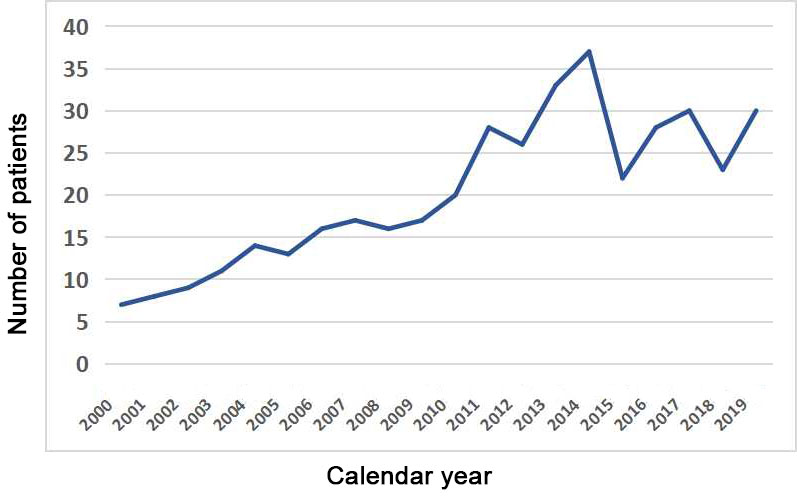

Supplement: Supplementary file 2 — Figure S2. [file CAM4-12-19523-s002.jpg]
