# Supplementary material for: Elderly nasopharyngeal carcinoma patients (aged ≥70 years): Survival and treatment strategies
Source: Cancer Med. 2023 Sep 19;12(19):19523–9. doi: 10.1002/cam4.6562 (PMC10587980; doi:10.1002/cam4.6562)
Supplement: Supplementary file 3 — Table S1. [file CAM4-12-19523-s001.doc]

**Supplemental table. Charlson Comorbidity Index**

| **score** | **Diseases** |
| --- | --- |
| **1** | Congestive heart failure |
|  | Myocardial infarction |
|  | Chronic obstructive pulmonary disease |
|  | Stomach ulcer disease  Peripheral vascular disease  Mild liver disease  Cerebrovascular disease  Connective tissue disease  Diabetes  Dementia |
| **2** | Hemiplegia |
|  | Moderate to severe chronic kidney disease  Diabetes with organ damage  Solid tumor  Leukemia  Malignant lymphoma |
| **3** | Moderate to severe liver disease |
| **6** | Metastatic solid tumor |
|  | Acquired immunodeficiency syndrome (AIDS) |
